# Targeting the WNT/β-Catenin Pathway in Hematological Malignancies: From Molecular Pathogenesis to Emerging Therapeutic Strategies

**DOI:** 10.3390/biom16050653

**Published:** 2026-04-28

**Authors:** Ali Keyhani, Hamed Haddad Kashani, Khadijeh Dizaji Asl, Zeinab Mazloumi, Faride Kaikavoosnejad, Seyyede Sepide Ashraf Moosavi, Milad Verdi, Ali Rafat, Reza Nejati

**Affiliations:** 1 Student Research Committee, Kashan University of Medical Sciences, Kashan, Iran; keyhani.ali799@gmail.com (A.K.); faride.kikavosnejad@gmail.com (F.K.); sepideh.amoosavi@gmail.com (S.S.A.M.); miladvrd@gmail.com (M.V.); 2 Anatomical Sciences Research Center, Institute for Basic Sciences, Kashan University of Medical Sciences, Kashan, Iran; hamedir2010@gmail.com; 3 Department of Histopathology and Anatomy, TaMS.C., Islamic Azad University, Tabriz, Iran; kh.asli2019@yahoo.com; 4 Department of Medical Applied Cell Sciences, Faculty of Advanced Medical Sciences, Tabriz University of Medical Sciences, Tabriz, Iran; zb.mazloumi@gmail.com; 5 Department of Pathology, Fox Chase Cancer Center, Temple University Health System, Philadelphia, PA 19111, USA

**Keywords:** WNT/β-catenin signaling, hematological malignancies, targeted therapy, combination therapy

## Abstract

Hematological malignancies, including multiple myeloma (MM), leukemia, and lymphoma, represent a major global health burden, accounting for approximately 6.6% of all cancer cases and contributing to significant mortality. The evolutionary conserved WNT/β-catenin signaling pathway is a critical regulator of normal hematopoietic stem cell homeostasis, and its dysregulation is a hallmark of various hematological malignancies. Aberrant activation through mutations, overexpression of ligands, or disruption of the destruction complex drives uncontrolled proliferation, impaired differentiation, and therapeutic resistance to therapy in acute and chronic leukemias, lymphomas, and multiple myeloma. Therapeutic interventions targeting this pathway, such as GSK-3 inhibitors, β-catenin antagonists, and small molecules like CWP291 and salinomycin, have demonstrated promising antitumor effects. Furthermore, combining WNT/β-catenin inhibition with targeted or epigenetic therapies, such as venetoclax and chidamide, can produce synergistic antitumor effects and overcome chemoresistance. Despite this potential, clinical translation is hampered by on-target toxicities in healthy tissues, pathway complexity, and a lack of predictive biomarkers. We conclude that the future of WNT-directed therapy lies in developing biomarker-selective agents, advanced drug delivery systems to improve specificity, and exploring novel combinations with immunotherapy to harness the anti-tumor immune response.

## 1. Introduction

Neoplastic disorders of the hematopoietic system encompass a range of diseases, including various forms of multiple myeloma, lymphomas, and leukemia [1]. Globally, hematological malignancies represent the fifth most frequently diagnosed group of cancers, accounting for approximately 6.6% of all cancer cases and 7.2% of cancer-related mortality in 2022 [2,3]. Genetic, biological, and physicochemical determinants significantly contribute to the pathogenesis of these malignancies. For instance, individuals exposed to formaldehyde or ionizing radiation exhibit a 20- to 30-fold increased risk of developing leukemia compared with the general population [1].

With ongoing societal development, the incidence of hematological malignancies continues to rise [4]. Despite progress in prevention, screening, and therapy, limited durable treatment options remain a major barrier to curing for many patients. As a result, substantial research efforts have focused on developing therapies that selectively target components of oncogenic signaling pathways, including the WNT/β-catenin axis, which has become a central focus in recent years [5,6].

The WNT signaling pathway regulates homeostasis and tissue development. This cascade is activated by the interaction of WNT proteins, lipid-modified secreted glycoproteins, with specific receptors and co-receptors. These signals then drive cellular processes such as invasion, differentiation, migration, polarity, apoptosis, and proliferation [7,8]. The WNT pathway comprises two major branches: the β-catenin-dependent (canonical) and β-catenin-independent (noncanonical) pathways [9]. Biochemical modulation of the canonical WNT pathway primarily influences cellular processes by modulating β-catenin levels and its intracellular distribution. Since the initial identification of the wingless-related integration site (WNT) in 1982, numerous studies have shown that the β-catenin-dependent pathway functions as a receptor-mediated signaling network with critical roles in the initiation, maintenance, and progression of diverse cancers [10,11].

Tumorigenesis and therapeutic resistance are frequently associated with dysregulation of critical signaling components. The WNT/β-catenin pathway, in particular, has gained considerable attention for its impact on cellular regulation and physiology [9]. Growing evidence indicates that abnormal WNT signaling contributes to hematological malignancy by altering stem cell dynamics, enhancing proliferation, and enabling resistance to therapy. This review focuses on the role of the WNT/β-catenin signaling pathway in the formation and progression of hematological malignancies. By integrating recent studies, we highlight the therapeutic potential of targeting this pathway, either alone or in combination with other treatments. This review aims to synthesize the current landscape of WNT/β-catenin signaling in hematological malignancies, critically evaluate the arsenal of therapeutic agents under development, and discuss innovative combination paradigms. Furthermore, we address the pivotal challenges of toxicity and resistance, offering perspectives on how future research guided by biomarker discovery and advanced technologies can unlock the full clinical potential of targeting this pivotal pathway in hematological oncology.

## 2. Canonical and Non-Canonical WNT/β-Catenin Signaling Pathway

The WNT signaling system is important for both normal physiology and disorders, such as cancer. In regular physiological processes, WNT proteins function as growth factors, regulating cell proliferation, tissue formation, and tissue organization, particularly through their influence on stem cells [12]. However, inappropriate activation is a major contributor to a variety of malignancies. A link was established between aberrant WNT pathway activity and the development of colorectal cancer (CRC) about 30 years ago. Since then, extensive studies have shown that disruption of the WNT signaling pathway is a characteristic hallmark in many malignancies, emphasizing its critical role in carcinogenesis. Mutations and aberrant activation of WNT components can lead to unregulated survival and cell proliferation, resulting in tumor initiation, development, and metastasis. For example, mutations in the β-catenin or adenomatous polyposis coli (APC) gene are commonly seen in CRC, leading to persistent activation of the WNT pathway. Additionally, abnormal WNT signaling has been linked to malignancies of the gastric, liver, and breast, among others [13].

The WNT signaling pathway is a highly conserved signaling cascade that governs fundamental cellular processes, including cell proliferation, apoptosis, differentiation, invasion, polarity, and migration [14]. This pathway plays a critical role in both embryonic development and tissue homeostasis throughout adulthood. Given these significant roles, dysregulation of WNT signaling has been implicated in numerous human diseases, most notably cancers [15].

Activation of the pathway primarily begins with the binding of WNT ligands to Frizzled (FZD) receptors. WNT proteins are lipid-modified secreted glycoproteins comprising 19 members in mammals [6]. Each FZD protein functions as a seven-pass transmembrane receptor, featuring a cysteine-rich domain (CRD) containing approximately ten cysteine residues, which is necessary for interacting with WNT ligands [6,16,17,18]. Beyond FZD, co-receptors such as low-density lipoprotein receptor-related proteins 5 and 6 (LRP5/6), receptor tyrosine kinase-like orphan receptors (ROR1 and ROR2), protein tyrosine kinase 7 (PTK7), and receptor-like protein tyrosine kinase (RYK), contribute to WNT recognition and signal propagation [6].

The WNT pathway is partitioned into β-catenin–independent (noncanonical) and β-catenin-dependent (canonical) routes, depending on the agonists, receptors, and intracellular components involved [1]. Certain WNT ligands, such as WNT1, WNT3A, and WNT8 bind to FZD receptors coupled with LRP5/6 co-receptors to activate canonical WNT signaling [14]. Conversely, WNT11 and WNT5A interact with ROR1, ROR2, and RYK receptors and to participate in the β-catenin–independent pathway [19].

Regardless of which signaling route is activated when WNT ligands bind to FZD or another receptor, the signal is typically transmitted to a central mediator known as Disheveled (DVL), which serves as a molecular hub for both canonical and noncanonical pathways [6]. DVL comprises an N-terminal DIX domain, a central PDZ domain, and a C-terminal DEP domain. These conserved domains mediate interactions with effector proteins that contribute to numerous signaling cascades [20]. The DIX and PDZ domains are implicated in activating β-catenin signaling, while the DEP and PDZ domains interact with proteins such as Daam1 and Prickle to activate β-catenin–independent signaling [6].

**Figure 1:**
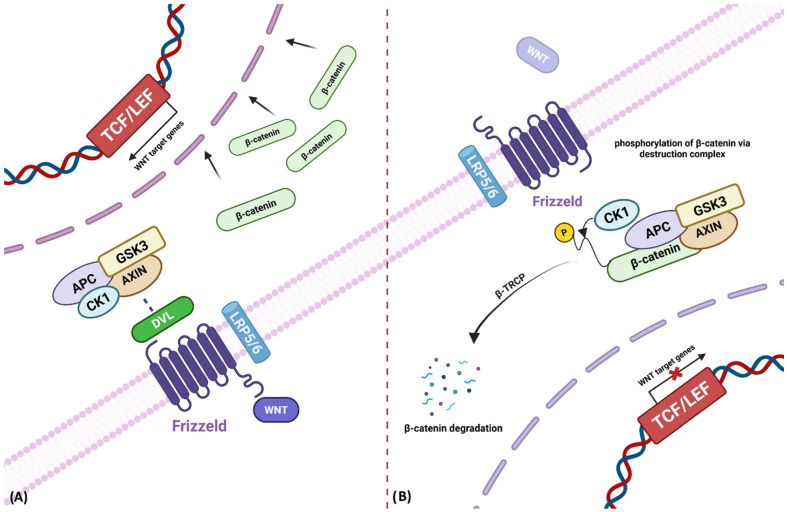
Canonical WNT/β-catenin signaling pathway. (**A**) Depicts the active state, where WNT ligands engage FZD and LRP5/6 co-receptors, activating Disheveled (DVL) and disrupting the β-catenin destruction complex (AXIN, APC, CK1, and GSK3). Stabilized β-catenin accumulates in the cytosol and translocates to the nucleus to associate with TCF/LEF transcription factors, driving expression of WNT target genes such as MYC, CCND1 (Cyclin D1), and BIRC5 (Survivin). (**B**). Illustrates the inactive state in the absence of WNT ligands, where β-catenin is phosphorylated and recognized by β-TrCP, followed by proteasomal degradation. This prevents nuclear translocation and transcriptional activation, thereby maintaining cellular homeostasis and preventing malignant transformation. Created in BioRender. keyhani, A. (2026) https://BioRender.com/vsvc4kr (accessed on 9 April 2026).

In the β-catenin-dependent pathway, in the absence of WNT ligands, the β-catenin destruction complex keeps the pathway in an “off” state [6] (Figure 1). This complex comprising Axin, casein kinase 1 (CK1), glycogen synthase kinase-3 (GSK3), and adenomatous polyposis coli (APC), phosphorylates β-catenin. The phosphorylated β-catenin is then recognized by the β-TRCP ubiquitin ligase and subsequently targeted for ubiquitination and proteasomal degradation [1]. This continual degradation prevents β-catenin accumulation and suppresses the transcription of WNT-responsive genes. Upon WNT ligand binding to FZD and LRP5/6, the pathway is activated, leading to LRP6 phosphorylation and the recruitment of DVL to the receptor complex [21,22]. Activated DVL facilitates the translocation of AXIN1 to the plasma membrane, resulting in the disassembly of the destruction complex. As a result, β-catenin becomes stabilized and accumulates in the cytoplasm before translocating into the nucleus, where it forms complexes with transcription factors TCF/LEF to drive the transcription of target genes such as Myc, Cyclin D1, Survivin, and MMPs, which are associated with the control of cell proliferation [23] (Figure 1).

**Figure 2:**
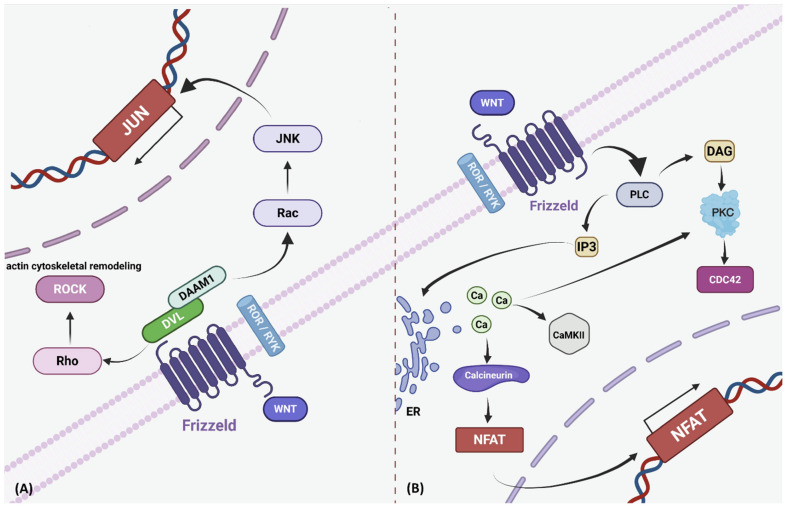
Non-canonical WNT signaling pathways: PCP and WNT/Ca^2+^ branches. (**A**) The planar cell polarity (PCP) pathway is initiated by WNT ligands binding to Frizzled (FZD) receptors and co-receptors such as ROR1/RYK, leading to activation of Disheveled (DVL). DVL engages DAAM1 to stimulate Rho and ROCK, driving actin cytoskeletal remodeling, while also promoting Rac and JNK activation, culminating in enhanced JUN-mediated transcription. (**B**) The WNT/Ca^2+^ pathway involves WNT–FZD interaction and activation of phospholipase C (PLC), which hydrolyzes membrane phospholipids to inositol 1,4,5-trisphosphate (IP3) and diacylglycerol (DAG). IP3 triggers Ca^2+^ release from the endoplasmic reticulum, activating CaMKII and calcineurin. Concurrently, DAG and Ca^2+^ activate protein kinase C (PKC), which stimulates Cdc42 to regulate cytoskeletal organization. Calcineurin and CaMKII promote dephosphorylation and nuclear translocation of NFAT, where NFAT modulates transcription related to cell fate and migration. Created in BioRender. keyhani, A. (2026) https://BioRender.com/6pxhfwx (accessed on 9 April 2026).

Furthermore, WNT ligands, such as WNT5A and WNT11, interact with receptors such as ROR1 and ROR2 and activate alternative signaling cascades, collectively referred to as noncanonical WNT pathways. The Planar Cell Polarity (PCP)/c-Jun N-terminal kinase (JNK) signaling pathway constitutes the first noncanonical route. This pathway activates proteins such as Rac, JNK, Rho, and Rho-associated kinase (ROCK) [24]. In this context, DVL interacts with DAAM1 to activate the small GTPase RhoA, which subsequently stimulates ROCK. This sequence facilitates actin cytoskeletal remodeling. Additionally, DVL promotes Rac1 activation, which leads to JNK activation. Activated JNK subsequently modulates actin dynamics, influencing cytoskeletal architecture and cell migration [10] (Figure 2).

The WNT/Ca^2+^ signaling pathway, another noncanonical branch that operates in conjunction with the PCP pathway, responds to WNT–Frizzled interactions. When the Frizzled receptor engages a ligand, it activates phospholipase C (PLC), which hydrolyzes phosphatidylinositol 4,5-bisphosphate (PIP2) into diacylglycerol (DAG) and inositol 1,4,5-trisphosphate (IP3) [25]. IP3 triggers Ca^2+^ release from the endoplasmic reticulum (ER), resulting in elevated cytosolic Ca^2+^ levels. DAG and Ca^2+^ activate protein kinase C (PKC), which subsequently activates Cdc42 to regulate actin polymerization, cellular migration, and polarization [25]. Elevated Ca^2+^ also activates Ca^2+^/calmodulin-dependent protein kinase II (CaMKII) and calcineurin, which regulate the nuclear factor of activated T cells (NFAT) through dephosphorylation and translocation [25]. In the nucleus, NFAT drives transcriptional programs controlling motility, morphogenesis, and cell fate. CaMKII also activates Nemo-like kinase (NLK), which antagonizes canonical WNT/β-catenin signaling, thereby maintaining the balance between canonical and noncanonical WNT activities [25] (Figure 2).

Other regulators modulate WNT signaling to fine-tune pathway activity. Norrin and the R-spondin (RSPO) family are prominent modulators. RSPO1–RSPO4 amplify both noncanonical and canonical WNT signaling. Norrin binds to FZD4 and stimulates canonical signaling [26].

WNT antagonists are broadly categorized into two groups: those that sequester WNT ligands and those that bind to the receptor complex. The first group includes secreted Frizzled-related proteins (sFRPs) and WNT inhibitory factor 1 (WIF-1). The sFRP family (sFRP1–sFRP5) binds various WNT ligands and inhibits both noncanonical and canonical signaling. WIF-1 directly binds WNT ligands, inhibiting both signaling axes [26]. The second category comprises the Dickkopf (DKK) family (DKK1–DKK4). DKK1, DKK2, and DKK4 bind to LRP5/6 to inhibit canonical signaling; DKK3 does not appear to bind LRP5/6 and may act via alternative pathways, such as TGF-β signaling [26].

**Figure 3:**
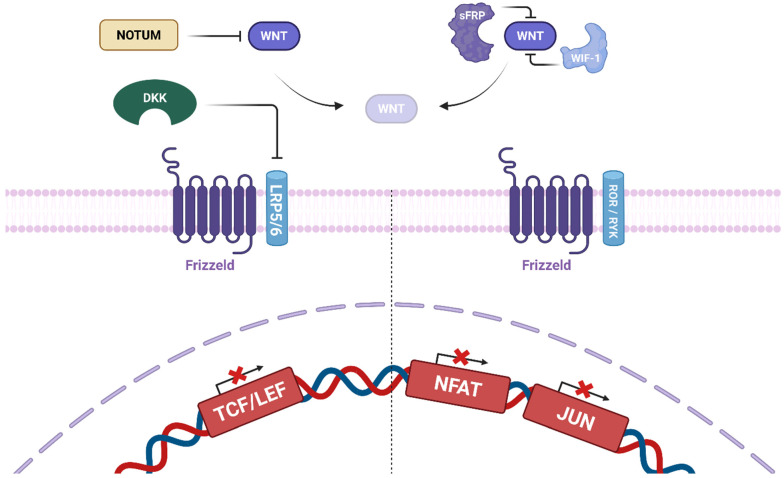
WNT-targeting strategies at the molecular level. The WNT signaling cascade in critical stages that can be targeted for therapeutic intervention. Extracellularly, WNT ligand activity is regulated by factors like NOTUM, which inactivates WNT proteins, and secreted antagonists such as sFRPs and WIF-1, which impede receptor interaction. At the membrane level, WNT signaling is mediated by Frizzled receptors and co-receptors such as LRP5/6 and ROR/RYK, with DKK family inhibitors interfering with receptor complex formation. Created in BioRender. keyhani, A. (2026) https://BioRender.com/flz06yi (accessed on 9 April 2026).

Additionally, other regulatory proteins such as Wise (SOSTDC1), Cerberus (CER1), and Kremen (KREM) can modulate WNT/β-catenin signaling. NOTUM functions as a deacylase, inactivating WNT ligands and thereby inhibiting pathway activity (Figure 3). To maintain precise control of signaling responses, several negative feedback mechanisms exist, including the regulation of WNT target genes such as AXIN1, AXIN2, SFRP, and DKK1, creating tightly controlled signaling dynamics [19].

## 3. Emerging Biomarkers in WNT/β-Catenin Signaling Cascade

Emerging biomarkers within the WNT/β-catenin signaling pathway such as WNT6, RNF43/ZNRF3, and R-Spondin family members have become increasingly important for characterizing cancer stemness, therapeutic resistance, and potential molecular targets, particularly in colorectal, hepatic, and pancreatic malignancies. Alongside nuclear β-catenin accumulation, these indicators contribute to predicting tumor responsiveness to WNT-directed therapeutic strategies [27,28].

WNT6 is notably associated with enhanced proliferative capacity in human colorectal cancer cell lines by accelerating cell-cycle progression [29]. Loss of WNT6 protein suppresses caspase-3 expression while upregulating the pro-apoptotic B-cell lymphoma 2-associated X protein (Bax), a key member of the Bcl-2 family. Conversely, WNT6 overexpression reverses these effects, demonstrating that WNT6 modulates apoptosis-related gene expression to inhibit programmed cell death and facilitate tumor progression. Elevated WNT6 levels also increase matrix metalloproteinase-2 (MMP2) expression, thereby promoting CRC cell migration [30].

The E3 ubiquitin ligases RNF43 and ZNRF3 function as negative regulators of WNT signaling by reducing cell-surface levels of FZD receptors. Loss-of-function mutations in these genes elevate the abundance of WNT receptors on the plasma membrane, sensitizing tumors to WNT-targeted therapies [31]. Although discovered more recently than other WNT pathway negative feedback regulators, RNF43 and ZNRF3 play essential roles in early embryonic development and tumorigenesis. They also interact with multiple components of both canonical and non-canonical WNT pathways, highlighting their central regulatory function [31].

R-Spondin family members (RSPO1-4) also modulate WNT/β-catenin signaling, with gene fusions or amplifications markedly enhancing pathway activation. Such alterations serve as clinically relevant biomarkers for predicting responsiveness to Porcupine inhibitors. RSPO proteins act cooperatively with WNT ligands to potentiate β-catenin signaling and have been detected across various human cancers, including ovarian, pancreatic, colorectal, breast, and lung malignancies [32].

## 4. Role of WNT/β-Catenin Signaling in Hematological; Malignancies

The WNT/β-catenin pathway regulates hematopoietic stem and progenitor cell (HSPC) function, though its disruption is linked to hematological malignancies. Inappropriate activation of this system alters the balance between self-renewal and differentiation, resulting in impaired hematopoietic homeostasis and malignant progression. Excessive activation and improper repression of WNT signaling have been linked to impaired stem cell function and altered long-term repopulation capacity, indicating a context-dependent involvement in the pathogenesis of the disease [19,33].

### 4.1. The WNT/β-Catenin Signaling Pathway in Leukemia

Extending from its critical role in normal hematopoiesis, the WNT/β-catenin signaling pathway has also been implicated in leukemogenesis.

Leukemias are malignant disorders arising from hematopoietic stem cells. Although leukemic cells proliferate excessively and suppress normal hematopoiesis, they typically arrest at immature stages due to dysregulated proliferation, impaired differentiation and apoptosis, and loss of normal cellular functions [34].

Leukemias are categorized as acute or chronic based on the degree of maturation and differentiation. Acute leukemia includes acute lymphoblastic leukemia (ALL) and acute myeloid leukemia (AML) while chronic leukemia encompasses chronic myeloid leukemia (CML) and chronic lymphocytic leukemia (CLL) [34]. Across these categories, aberrant activation of the WNT/β-catenin pathway, driven by dysregulated ligand expression or defects in the destruction complex, contributes to leukemogenesis [34,35].

In this context, WNT signaling is particularly prominent in T-ALL, with evidence showing that a substantial proportion of pediatric patients exhibit elevated β-catenin levels and upregulated expression of WNT target genes such as LEF, AXIN2, TCFL, and c-Myc [36]. Additionally, approximately 30% of adult T-ALL patients have high levels of lymphoid enhancer-binding factor 1 (LEF1) mRNA, which promotes cell proliferation and survival by activating WNT target genes and recruiting of β-catenin [36].

Similarly, aberrant regulation of the WNT/β-catenin pathway has been associated with B-ALL, as exemplified by a pre-B ALL subtype characterized by t(1;19) translocations. In this subtype, WNT16b is markedly activated by the expression of the E2A-PBX1 fusion protein [37]. Moreover, Mazieres and colleagues reported elevated WNT16b alongside β-catenin, Dvl2, and TCF4 in cells carrying t(1;19), further indicating activation of the WNT/β-catenin pathway. Other studies have shown that WNT proteins promote B-ALL cell proliferation, and WNT/β-catenin signaling is crucial for B-ALL leukemia stem cell (LSC) survival within the bone marrow stromal niche [37]. Further supporting its pathogenic role, several studies have demonstrated upregulation of WNT components in primary AML samples, including overexpression of ligands such as WNT1, WNT2B, and WNT10B, transcription factors like LEF-1, and increased receptor levels such as FZD4 [38,39,40]. These alterations have been associated with enhanced resistance to apoptosis [38,39].

Chronic lymphocytic leukemia (CLL), one of the most common adult leukemias, also exhibits substantial dysregulation of this pathway [41]. Leukemogenesis in CLL involves genomic changes that disrupt the regulation of proliferation and apoptosis in clonal B cells [41].

Studies have shown that the WNT signaling pathway is aberrantly activated in CLL cells, and persistent WNT/β-catenin activity appears to play a key role in the defective apoptotic response characteristic of this malignancy [42]. In CLL cells, LEF-1 is significantly upregulated compared to normal blood B cells. Additionally, ROR1, whose promoter contains several LEF-1 regulatory motifs, is also overexpressed in CLL [42]. Chronic myeloid leukemia (CML) is a clonal hematopoietic stem cell malignancy defined by the presence of the BCR-ABL oncogene resulting from the t(9;22)(q34;q11) translocation [43]. This reciprocal translocation drives constitutive tyrosine kinase activity, promoting prolonged survival and uncontrolled proliferation of myeloid cells [43]. In CML, Disheveled (DVL) genes, which are normally silenced in healthy bone marrow, exhibit abnormal and heterogeneous expression patterns in patient bone marrow [43].

Experimental investigations show that modulating DVL expression in CML cell lines, whether by silencing or overexpression, significantly affects the expression of key signaling regulators, including SMAD1, AHR, mTOR, and BRD7, which govern cellular proliferation, differentiation, and survival [43].

Expanding on these findings, another study demonstrated in cell-based experiments that PLIN2 enhances the expression of AKT, β-catenin, p-AKT, GSK-3β, and Axin2 expression, promoting CML progression through combined activation of the GSK3 and WNT/β-catenin signaling pathways [44]. Moreover, they showed that CCAAT/enhancer-binding protein alpha (CEBPA)-mediated upregulation of PLIN2 stimulates tumor growth in vivo through the same signaling cascades [44]. Acute and chronic leukemias constitute a diverse group of aggressive malignancies with variable clinical outcomes. Despite advances in chemotherapy and targeted agents (e.g., BCR-ABL or FLT3 inhibitors), relapse and therapy resistance remain major obstacles, often driven by persistent leukemia stem cells (LSCs). The WNT/β-catenin pathway has emerged as a key regulator of LSC self-renewal and survival, making it a compelling therapeutic target for preventing relapses and overcoming resistance.

### 4.2. The Role of the WNT/β-Catenin Signaling Pathway in Lymphoma

Lymphomas are hematological malignancies arising in extranodal lymphoid tissues and lymph nodes, and they are histopathologically classified as Hodgkin lymphoma or non-Hodgkin lymphoma (NHL), each with distinct clinical and biological characteristics [45]. Across lymphomas, investigations of canonical WNT signaling in lymphoma have predominantly focused on diffuse large B-cell lymphoma (DLBCL) [1].

For instance, CircRNA-APC has been shown to suppress the proliferation of DLBCL cells by inhibiting WNT/β-catenin signaling. In gain-of-function studies, increased CircRNA-APC expression reduced tumor progression in vivo and DLBCL cell proliferation in vitro [46].

In line with these findings, a pair of closely related hub genes, FBN1 and TIMP1, were identified in DLBCL via differential expression analysis and bioinformatics evaluation. The FBN1/TIMP1 interaction promotes DLBCL cell migration and modulates WNT signaling [47].

Another study demonstrated that WNT-10A is a critical component of the WNT/β-catenin pathway, and its regulation by miR-361-3p can influence the molecular mechanisms underlying lymphoma development [48]. Aggressive subtypes like DLBCL and mantle cell lymphoma (MCL), can be refractory to standard immunochemotherapy, largely due to the tumor microenvironment and aberrant signaling pathways. Emerging evidence implicates dysregulated WNT/β-catenin signaling in promoting lymphoma cell proliferation, survival, and interaction with the niche, highlighting its potential as a novel target.

### 4.3. The WNT/β-Catenin Signaling Pathway in Multiple Myeloma

Multiple myeloma (MM) is a refractory hematological malignancy characterized by abnormal proliferation and accumulation of plasma cells. MM patients have an increased risk of chemotherapy resistance and relapse. The bone marrow microenvironment plays a pivotal role in MM progression. Within this milieu, stromal cells secrete WNT ligands that activate WNT signaling in MM via the transcription regulator β-catenin. The WNT/β-catenin pathway normally promotes osteoblast differentiation and bone formation, whereas dysregulation contributes to MM cell proliferation and drug resistance [9,49]. Multiple myeloma remains an incurable plasma cell malignancy characterized by inevitable relapse and the development of multidrug resistance. The protective bone marrow microenvironment plays a crucial role in fostering MM cell growth and chemoresistance. Within the protective bone marrow niche, WNT/β-catenin signaling acts as a critical mediator of myeloma-stromal interactions, osteolytic bone disease, and drug resistance, presenting a multifaceted target for therapy [9].

Collectively, studies suggest that WNT/β-catenin signaling plays a crucial role in the development, progression, and maintenance of hematopoietic malignancies. This pathway has emerged as a promising therapeutic target due to its role in controlling leukemic stem cell survival, proliferation, and therapy resistance. Pharmacological techniques to modulate WNT/β-catenin signaling have been developed, either alone or in combination with existing treatments, based on these observations.

## 5. WNT/β-Catenin Signaling Pathway Interventions for Hematological Malignancies

Radiotherapy and chemotherapy remain the most common conventional cancer therapies; however, their efficacy is limited. They are associated with substantial adverse effects, including bone marrow suppression and gastrointestinal toxicity. Consequently, there is a critical need to develop novel, more effective, and safer strategies for treating hematological malignancies. Several investigations indicate that inhibiting the WNT/β-catenin pathway can be beneficial in hematological cancers (Table 1).

Several studies are currently exploring therapeutic approaches in leukemia that target the WNT/β-catenin signaling axis. Notably, a Phase II trial reported that LY2090314, a glycogen synthase kinase-3 (GSK-3) inhibitor, exhibited a favorable safety profile in patients with acute myeloid leukemia (AML) [50]. These findings are consistent with observations from the initial Phase I trial in humans. Although β-catenin levels confirmed on-target activity of GSK-3 inhibition, none of the participants achieved complete or partial remission [50]. Only five of twenty patients continued therapy beyond two treatment cycles. LY2090314 was generally well tolerated in this population; overall, nausea, decreased appetite, dry mouth, fatigue, dyspepsia, and prolonged QT intervals were the most common potentially drug-associated treatment-emergent adverse events (TEAEs) [50]. Five of the seven patients who passed away during the research died during the follow-up period, and febrile neutropenia and syncope were potentially drug-related serious adverse events (SAEs) [50]. Both AE-related deaths, which were due to sepsis and cerebral hemorrhage were thought to be potentially drug-related. However, given the modest clinical benefit observed, its development as a monotherapy for this indication is not supported [50].

In a related Phase I trial involving relapsed or refractory AML and myelodysplastic syndromes (MDS), the small-molecule inhibitor CWP232291 (CWP291), which targets the WNT/β-catenin pathway, demonstrated a favorable safety and tolerability profile [51]. The most common treatment-emergent adverse events (TEAEs) were myalgia, infusion-related reactions, and gastrointestinal symptoms, specifically diarrhea, vomiting, and nausea. Gastrointestinal toxicities were seen at all dose levels, with no evident dose-related patterns. CWP291 was thus classified as marginally to moderately emetogenic; however, it could be effectively treated with basic antiemetics and regular prophylaxis [51]. Infusion-related reactions, such as paresthesia and myalgia, were successfully controlled with analgesics, antihistamines, and/or corticosteroids as needed, and resolved entirely with no sequelae in all instances. Additionally, prolonging the duration of the infusion from thirty minutes to two hours appeared to somewhat alleviate myalgia symptoms at the maximum tolerated dose (MTD) [51]. Although CWP291 showed limited efficacy as a single agent (one complete response and one partial response), pharmacodynamic analyses confirmed inhibition of the targeted pathway, evidenced by decreased plasma levels of β-catenin and survivin [51].

Beyond AML, targeting the WNT/β-catenin signaling axis has shown potential in treating acute lymphoblastic leukemia (ALL). Deferoxamine (DFO), an iron chelator, reduced tumor growth and proliferation, diminished reactive oxygen species production, and impeded cell cycle progression [52]. DFO also induced apoptosis in ALL cell lines by inhibiting WNT/β-catenin signaling, hypoxia-inducible factor-1α (HIF-1α)/prolyl hydroxylase 2 (PHD-2), and the p38 MAPK/ERK pathways via iron depletion [52].

Similarly, modulation of this pathway has been shown to influence disease progression in chronic lymphocytic leukemia (CLL). For instance, salinomycin, an antibiotic potassium ionophore, can selectively inhibit WNT-induced LRP6 phosphorylation and reduce WNT target gene expression, with preferential toxicity toward primary CLL cells relative to normal peripheral blood mononuclear cells (PBMCs) [53]. These findings imply that agents inhibiting LRP6 phosphorylation or stability may have therapeutic activity in CLL [53].

Additional studies in chronic myeloid leukemia (CML) and acute promyelocytic leukemia (APL) models support the therapeutic potential of WNT pathway disruption. The imidazole-derived compound L-7 induces apoptosis and inhibits cellular proliferation by suppressing WNT/β-catenin signaling, resulting in the downregulation of WNT target genes such as EYA3, Axin2, c-Myc, and AXL receptor tyrosine kinase [54], underscoring the broad applicability of WNT-inhibiting agents across leukemia subtypes.

Therapeutic strategies targeting the WNT/β-catenin pathway in lymphoma and multiple myeloma (MM) are actively being investigated, with encouraging preclinical findings. In lymphoma, the overexpression of POLE2 (DNA polymerase epsilon subunit 2) promotes tumor growth via activation of WNT/β-catenin signaling. Silencing POLE2 inhibits tumor growth and migration, induces apoptosis and cell-cycle arrest, and reduces proliferation in xenograft models, identifying POLE2 as a potential therapeutic target in lymphoma [55].

In MM, several approaches are under exploration. Among them, the Dujieqing (DJQ) decoction has demonstrated potent anti-myeloma activity [56]. DJQ-derived serum or decoction reduced the expression of Cyclin D1, c-Myc, LEF1, and β-catenin/LEF1 in xenograft models, resulting in decreased proliferation, enhanced apoptosis, and reduced tumor burden without detectable toxicity. DJQ presents as a promising natural therapeutic strategy for MM via inhibition of the β-catenin pathway [56].

Emerging targeted protein degradation modalities most notably proteolysis-targeting chimeras (PROTACs) represent a paradigm-shifting therapeutic strategy for addressing historically “undruggable” targets, including non-enzymatic scaffolding proteins such as β-catenin. As the most advanced and extensively characterized class of β-catenin-directed degraders, PROTACs function as heterobifunctional molecules that simultaneously engage the target protein and a cognate E3 ubiquitin ligase, thereby inducing proximity-driven polyubiquitination and subsequent degradation by the 26S proteasome [57,58].

Collectively, the WNT/β-catenin signaling axis has been widely explored across hematological malignancies, with therapeutic strategies ranging from small-molecule inhibitors to natural compounds and pathway-modulating biologics. Recent efforts have increasingly focused on integrating WNT pathway inhibition with other therapeutic modalities to enhance efficacy, overcome resistance, and improve clinical outcomes. A critical limitation of many preclinical studies summarized in Table 1 is their predominant reliance on established cell lines and xenograft models, which poorly recapitulate the native bone marrow microenvironment. Several studies lack orthogonal validation. Only a minority report negative controls for off-target effects.

**Table 1:** Studies Indicating the Therapeutic Potential of WNT/β-Catenin Pathway Inhibition in Hematological Malignancies.

Factor	Target	Disease	Model	Ref./Identifier
LY2090314	GSK-3	AML	Human (phase 2)	[50] NCT01214603
CWP232291	β-catenin	AML	Human (phase 1)	[51] NCT01398462
Compound 41	CTNNB1	AML	In vivo/in vitro	[59]
DACT3	DVL2	AML	In vivo/in vitro	[60]
NUC-7738	β-catenin	AML	in vitro	[61]
Zl-n-91	type 4 phosphodiesterase (PDE4)	AML	In vivo/in vitro	[62]
Matrine	miR-495-3p and miR-543	AML	In vitro	[63]
Deferoxamine	c-Myc, cyclinD1, and β-catenin	ALL	In vivo/in vitro	[52]
Erlotinib	Epidermal Growth Factor Receptor (EGFR)	T-ALL	In vivo/in vitro	[64]
Chidamide	histone deacetylase (HDACs)	B-ALL	In vivo/in vitro	[65]
imidazole	AXL-RTK, c-Myc, Axin 2, and EYA3	CML, APL	in vitro	[54]
Quercetin (Qu)	GSK-3β, β-catenin, Lef-1, PPAR-δ, Cyclin D1	CML	in vitro	[66]
siRNA	β-catenin, pLRP6	CML	in vitro	[43]
PRI-724	CBP/β-catenin	AML; CML	Human (Phase 2)	NCT01606579
miR-34a	WNT1	CLL	in vitro	[67]
ethacrynic acid (EA)	LEF-1	CLL	in vitro	[42]
Salinomycin	LRP6	CLL	in vitro	[53]
CGP049090 or PKF115-584	LEF-1	CLL	In vivo/in vitro	[68]
POLE2 knockdown	POLE2	Lymphoma	In vivo/in vitro	[55]
Salinomycin sodium	LRP6	Diffuse large B-cell lymphoma (DLBCL)	in vitro	[69]
FNC	β-catenin, MMP-2, MMP-9, and VEGF, along with upregulation of GSK-3β and E-cadherin	Non-Hodgkin Lymphoma (NHL)	in vitro	[70]
miR-361-3p	WNT10A	Lymphoma	in vitro	[48]
AV-65	β-TrCP	MM	In vivo/in vitro	[71]
GSK126	enhancer of zeste homolog 2 (EZH2), an epigenetic regulator	MM	In vivo/in vitro	[72]
Lycorine	β-catenin	MM	in vitro	[73]
miR-19a-3p	WNT1	MM	in vitro	[74]
Polyphyllin VII	Moesin (MSN)	MM	In vivo/in vitro	[75]
CGK012	β-catenin	MM	in vitro	[76]
Dujieqing (DJQ)	β-catenin	MM	In vivo/in vitro	[56]
siRNA	Ribonucleotide reductase M2 (RRM2)	MM	in vitro	[77]
Rubia philippinensis extract	β-catenin	MM	in vitro	[78]
BC2059	Transducin β-like protein 1 (TBL1) and its related protein (TBLR1)	MM	In vivo/in vitro	[79]
CKD-581	histone deacetylase (HDAC)	MM	In vivo/in vitro	[80]

## 6. Combination Therapy

A combination of therapies has emerged as a pivotal approach to improving treatment outcomes and overcoming resistance encountered with monotherapy. In the context of hematological malignancies, combinations pairing WNT/β-catenin signaling axis inhibitors with either conventional chemotherapeutics or targeted agents have been explored to achieve synergistic anticancer effects by concurrently modulating complementary cellular and molecular pathways (Table 2).

A recent study demonstrated that combining C-82, a WNT/β-catenin pathway inhibitor, with venetoclax (VEN), a highly selective BCL-2 inhibitor, synergistically suppresses AML cell proliferation and induces apoptosis [81]. Mechanistically, C-82 destabilizes MCL-1, with downregulation associated with multiple phosphorylation sites and proteasomal degradation. The combination of C-82 and VEN synergistically triggers mitochondrial apoptosis and gasdermin E (GSDME)-mediated pyroptosis [81].

Another study examined the combined effect of venetoclax and chidamide on MYCN/DKK3 regulation and the WNT/β-catenin pathway in B-ALL [82]. In vitro and in vivo findings showed that co-treatment with chidamide and venetoclax synergistically suppressed MYCN expression while upregulating DKK3 by inhibiting histone deacetylase (HDAC) and BCL2 activity [82]. This dual inhibition effectively attenuated WNT/β-catenin signaling and reduced B-ALL cell proliferation. The results suggest that the HDAC inhibitor chidamide, in combination with the BCL2-targeted venetoclax, could serve as a promising combinatorial therapeutic strategy for B-ALL [82].

In CML models, simvastatin impaired the survival of both imatinib-sensitive and -resistant CML cells, including those harboring the T315I mutation [83]. Co-administration of simvastatin with imatinib produced synergistic cytotoxic effects both in vitro and in vivo, via suppression of the PI3K/Akt pathway and downregulation of the canonical WNT/β-catenin pathway [83]. These molecular events were accompanied by reduced Myc expression and enhanced H3K27 trimethylation. Collectively, these findings suggest that combining simvastatin with imatinib may provide an effective strategy to overcome imatinib resistance in CML [83].

In mantle cell lymphoma (MCL), an aggressive form of non-Hodgkin lymphoma, concurrent activation of the Hedgehog/GLI1 and WNT/β-catenin pathways promotes enhanced cellular proliferation and therapeutic resistance [84]. Treatment combining GANT61 (GLI1 inhibitor) with ICG-001 (β-catenin/CBP inhibitor) yielded a synergistic reduction in MCL cell viability, induced G0/G1 cell-cycle arrest, and promoted apoptosis through downregulation of GLI1, β-catenin, Cyclin D1, and BCL-2, with upregulation of GSK-3β and p21 [84]. CBP (CREB-binding protein) and β-catenin physically associate to form a transcriptional co-activator complex that amplifies the expression of genes central to oncogenesis, stem cell self-renewal, and pathological fibrosis. This partnership directs chromatin occupancy at enhancers and super-enhancers, particularly those responsive to WNT signaling, thereby potentiating transcription of downstream WNT target genes [85,86]. Furthermore, dual inhibition increased the sensitivity of MCL cells to doxorubicin and the Bruton’s tyrosine kinase (BTK) inhibitor ibrutinib, suggesting that concurrent targeting of both pathways may offer an effective therapeutic strategy for MCL [84].

Polyphyllin VII (PP7), a bioactive compound derived from Rhizoma Paris, has been shown to preferentially inhibit the proliferation of multiple myeloma (MM) cells in vitro and in vivo [75]. Moesin, a key regulator of the WNT/β-catenin signaling pathway, was identified as a molecular target of PP7 via drug affinity responsive target stability and cellular thermal shift assays [75]. The binding of PP7 to moesin promoted its ubiquitin-proteasome–mediated degradation, thereby diminishing WNT/β-catenin pathway activity and reducing the proportion of side population cells, contributing to overcoming bortezomib resistance. Overall, these findings position polyphyllin VII as a promising therapeutic agent and moesin as a potential biomarker and treatment target for MM [75].

While combination strategies targeting the WNT/β-catenin pathway have shown encouraging preclinical outcomes across various hematological malignancies, several challenges remain that hinder full clinical translation. Notably, most combination studies have been conducted in vitro or in xenograft models with limited assessment of additive versus synergistic effects using rigorous statistical methods (e.g., Chou-Talalay combination index). Moreover, none of the cited combination studies provide formal evaluation of whether the observed synergy is WNT/β-catenin-dependent or arises from off-target pathway modulation.

Therefore, a rigorous understanding of the pharmacological, biological, and micro environmental factors associated with WNT inhibition is essential to optimize these therapeutic strategies.

Rationale for Combination Strategies: The synergy observed in combining WNT/β-catenin inhibitors with other agents stems from several key mechanisms: (1) Vertical Pathway Blockade: Simultaneously targeting upstream (e.g., ligand secretion) and downstream (e.g., β-catenin/TCF transcription) components can enhance pathway suppression. (2) Horizontal Pathway Crosstalk: Inhibiting WNT alongside parallel survival pathways (e.g., PI3K/AKT, BCL-2) can overcome compensatory signaling and bypass resistance. (3) Microenvironment Disruption: Combining WNT inhibitors with drugs that target the protective niche (e.g., stromal cells, immune modulators) can displace malignant cells from their supportive environment. However, the primary challenge remains the balance between enhanced efficacy and potentially compounded toxicities, necessitating careful preclinical modeling and biomarker-driven patient stratification in clinical trials.

**Table 2:** Studies Indicating the Therapeutic Potential of WNT/β-Catenin Pathway Inhibition with either conventional chemotherapeutics or targeted agents in Hematological Malignancies.

Factors	Type of Combination	Disease	Model	Ref.
C-82 + VEN	Targeted therapy (Bcl-2 inhibitor Venetoclax) + WNT/β-catenin pathway inhibition(C-82)	AML	In vivo, In vitro	[81]
Dinaciclib + PLX51107	Targeted therapy (BET inhibitor PLX51107) + WNT/β-catenin pathway inhibition (CDK inhibitor Dinaciclib)	AML	In vivo, In vitro	[87]
Compound 31 + idarubicin	Chemotherapy (Idarubicin) + WNT/β-catenin inhibitor (compound 31)	AML	In vitro	[88]
(Sorafenib/Quizartinib) + (C-82/PRI-724)	Targeted therapy (FLT3 tyrosine kinase inhibitor [Sorafenib/Quizartinib]) + WNT/β-catenin pathway inhibition (β-catenin/CBP inhibitor [C-82/PRI-724])	AML	In vivo, In vitro	[89]
salinomycin + all-trans retinoic acid (ATRA)	Differentiation therapy (All-trans Retinoic Acid) + WNT/β-catenin pathway inhibition (Salinomycin)	AML	In vitro	[90]
BC2059 + Panobinostat	Epigenetic therapy (HDAC inhibitor Panobinostat) + WNT/β-catenin pathway inhibition (β-catenin antagonist BC2059)	AML	In vivo, In vitro	[91]
Ara-C or Idarubicin + WNT/GSK-3 inhibitors	chemotherapy (Ara-C/Idarubicin) + WNT/β-catenin pathway inhibition (Niclosamide, LiCl, PNU-74654, or AR-A014418)	AML	In vivo, In vitro	[92]
Chidamide + venetoclax	Epigenetic therapy (HDAC inhibitor Chidamide) + Targeted apoptosis therapy (BCL-2 inhibitor Venetoclax) → Indirect suppression of the WNT/β-catenin pathway via MYCN/DKK3	B-ALL	In vivo, In vitro	[82]
DKK1 + Vincristine (VCR), Vindesine (VDS), Etoposide (VP-16), Doxorubicin, Prednisolone	Chemotherapy (VCR, VDS, VP-16, Doxorubicin, Prednisolone) + WNT/β-catenin pathway inhibition (DKK1)	pediatric ALL	In vitro Ex vivo	[93]
Simvastatin + Imatinib	Targeted therapy (BCR-ABL inhibitor Imatinib) + Metabolic modulator (HMG-CoA reductase inhibitor Simvastatin) → indirect WNT/β-catenin pathway inhibition	CML	In vivo, In vitro	[83]
AZT + Emodin	Telomerase inhibition (AZT) + WNT/β-catenin pathway inhibition (Emodin)	CML	In vitro	[94]
TCF7 knockdown + Imatinib	Gene-targeted therapy + Targeted therapy (TCF7 knockdown–mediated WNT/β-catenin pathway inhibition combined with the BCR-ABL inhibitor Imatinib)	CML	In vitro	[95]
C82/PRI-724 + Nilotinib	Targeted therapy (BCR-ABL tyrosine-kinase inhibitor [TKI] Nilotinib) + WNT/β-catenin pathway inhibition (C82 or PRI-724)	BC-CML	in vitro, ex vivo, and in vivo	[96]
GANT61 and ICG-001+ doxorubicin/ibrutinib	Dual signaling pathway inhibition (Hedgehog/GLI1 inhibitor GANT61 + WNT/β-catenin inhibitor ICG-001) Additional assessments with Chemotherapy (doxorubicin) and Targeted therapy (ibrutinib) combinations	MCL	in vitro, ex vivo	[84]
PFI-1 + CPI-203	Dual epigenetic inhibition (BET inhibitors PFI-1 and CPI-203)	follicular lymphoma	in vitro, ex vivo	[97]
Polyphyllin VII + Bortezomib	Proteasome inhibitor/chemotherapeutic agent (Bortezomib) + WNT/β-catenin pathway inhibition (Polyphyllin VII)	MM	In vivo, In vitro	[75]
ICG-001/Pyrvinium + Bortezomib	Proteasome inhibitor/chemotherapeutic agent (Bortezomib) + WNT/β-catenin pathway inhibition (β-catenin inhibitors ICG-001 or Pyrvinium)	MM	in vitro	[98]
Decitabine + Bortezomib	WNT/β-catenin pathway inhibition (Decitabine) + Proteasome inhibitor/chemotherapeutic agent (Bortezomib)	MM	In vivo, In vitro	[99]
BC2059 + Bortezomib	Proteasome inhibitor/chemotherapeutic agent (Bortezomib)+ WNT/β-catenin pathway inhibition (β-catenin inhibitor BC2059)	MM	in vitro, ex vivo, and in vivo	[79]

## 7. Challenges and Future Perspectives

The clinical efficacy of therapies targeting the WNT signaling pathway remains suboptimal, presenting substantial therapeutic challenges [11]. Although WNT/β-catenin–targeted interventions are diverse, clinical experience with these agents remains limited. While the development of novel targeted therapies and combination strategies is actively being pursued, risks of toxicity, adverse effects, and off-target activity cannot be ignored [17,100].

The WNT/β-catenin signaling pathway plays a critical role in osteogenesis, tissue regeneration, hepatic homeostasis, hair follicle cycling, and cardiac development, in addition to its involvement in tumorigenesis [101,102,103,104]. Consequently, clinical trials frequently report on-target, off-tumor toxicities. These adverse events (AEs) primarily affect bone health, hematopoietic function, hair, and the gastrointestinal tract, thereby limiting the practical use of WNT-targeted therapeutic options [11].

Furthermore, the WNT axis interacts with multiple signaling cascades, including EGFR, PI3K/AKT/mTOR, STAT3, and NF-κB. These intricate ligand–receptor interactions are essential for fundamental physiological processes, and disruption can precipitate severe AEs [105,106,107,108].

However, it is important to note that much of the evidence for such crosstalk comes from solid tumors (e.g., colorectal, lung, and gastric cancers), with limited direct validation in hematological malignancies. Therefore, extrapolation to leukemias, lymphomas, and multiple myeloma requires caution.

Therefore, targeting canonical WNT signaling may yield unintended consequences given its broad role in regulating normal tissue homeostasis [109].

Redundancy within the WNT signaling pathway including its numerous ligands, receptors, and downstream effectors plays a critical role in promoting therapeutic resistance across cancers. Because this pathway is highly conserved and multifunctional, inhibiting a single component often triggers compensatory mechanisms that maintain tumor survival and self-renewal. Cancer cells frequently express diverse WNT ligands and FZD receptors, enabling continuous β-catenin activation even when specific elements are blocked [110]. Functional redundancy also preserves cancer stem cell properties, contributing to recurrence. Moreover, WNT-driven tumors can bypass pharmacological inhibition by activating parallel signaling routes or alternative WNT-activating factors. Extensive crosstalk with pathways such as Hedgehog, Notch, and TGF-β further reinforces resistance, while the presence of multiple co-receptors limits the effectiveness of targeted inhibitors [110,111].

Additionally, systemic distribution of agents can incur unexpected toxicities in healthy tissues. Liposomal drug delivery can enhance tumor-specific localization while reducing systemic exposure [112]. Nevertheless, WNT/β-catenin inhibitors, whether used as monotherapy or in combination with other anticancer agents, have been associated with a range of AEs. The most common adverse effects include nausea, diarrhea, vomiting, hemorrhage, fatigue, bone marrow suppression with neutropenia, fractures, and headache [5]. Additionally, due to patient frailty and comorbidity, dose escalations are sometimes curtailed before reaching optimal therapeutic concentrations, leading to inconsistent data regarding anticancer efficacy of examined WNT/β-catenin inhibitors [5].

WNT-targeted therapies, like many targeted modalities, show efficacy that is contingent on specific molecular phenotypes. For example, PORCN inhibitors primarily exhibit activity in RSPO3 fusions and RNF43-mutant tumors, whereas DKN-01 is effective only in patients with elevated Dkk1 expression. This phenotype-dependent specificity limits the broader applicability of these targeted treatments [113,114].

A more rigorous evaluation of existing WNT/β-catenin inhibitors will be essential for overcoming the current barriers to developing effective therapies for hematological malignancies. Furthermore, advanced approaches such as synergistic combination regimens and immunotherapy may yield substantial advances toward identifying effective WNT/β-catenin–targeted therapies in oncology [109,115]. Future Perspectives: To overcome the current limitations, future efforts should focus on several strategic fronts: (1) Biomarker-Driven Therapy: Identifying and validating predictive biomarkers (e.g., specific mutations, RSPO fusions, DKK1 expression) is paramount for selecting patients most likely to benefit from WNT-targeted drugs, advancing the goal of personalized medicine. (2) Advanced Delivery Systems: Utilizing nanoparticle carriers, antibody-drug conjugates (ADCs), or dual-targeting ligands can improve tumor-specific delivery of WNT inhibitors, maximizing on-target effects while minimizing systemic toxicity to healthy tissues like bone and gut. (3) Immunotherapy Combinations: Given the role of WNT signaling in creating an immunosuppressive tumor microenvironment, combining WNT inhibitors with immune checkpoint blockers (e.g., anti-PD-1), CAR-T cells, or bispecific antibodies represents a promising strategy to restore anti-tumor immunity and achieve more durable clinical responses. (4) Next-Generation Agents: Developing allosteric inhibitors, protein degradation technologies (e.g., PROTACs targeting β-catenin), and isoform-specific agents may offer improved selectivity and safety profiles over current compounds.

To improve clinical translation in hematological malignancies, existing WNT/β-catenin inhibitors must be thoroughly evaluated. Future studies should focus on the development of more selective and particular to the setting modulators, as well as biomarker-driven approaches to identifying patients who will benefit most from WNT-targeted medicines. Advanced methods, such as combining WNT/β-catenin inhibitors with targeted medicines, epigenetic treatments, or immunotherapies, can improve therapeutic efficacy and overcome resistance mechanisms. Integrating molecular profiling with translational investigations is crucial for improving and developing WNT/β-catenin-targeted treatments in hematological oncology.

## 8. Conclusions

In conclusion, the WNT/β-catenin signaling pathway constitutes a crucial regulatory axis in hematological malignancies, influencing disease progression and therapeutic response. This review provides a comprehensive synthesis of the molecular architecture of canonical and noncanonical WNT signaling, with particular emphasis on its roles in leukemia, lymphoma, and multiple myeloma. Dysregulation of β-catenin activity, including ligand overexpression and disruption of degradation complexes, has been linked to malignant progression and treatment resistance. Therapeutic strategies targeting this pathway, such as GSK-3 inhibitors, β-catenin antagonists, and natural compounds, have demonstrated potential in both preclinical and clinical settings. Moreover, combination therapies that pair WNT inhibitors with agents like venetoclax, chidamide, or imatinib have shown synergistic efficacy in overcoming resistance. Nevertheless, the broad physiological effects of WNT/β-catenin signaling raise safety concerns, as off-target toxicity and pathway crosstalk continue to limit clinical applicability. Although targeting WNT/β-catenin remains a promising therapeutic strategy, future efforts must refine pathway specificity, improve delivery approaches, and mitigate adverse events to enable its effective use in hematological malignancies.

## Data Availability

Data is contained within the article.

## References

[1] Yu S., Han R., Gan R. (2022). The Wnt/β-catenin signalling pathway in Haematological Neoplasms. Biomark. Res..

[2] Munir M., Cheema A.Y., Kakakhel M.Z.J., Sinha S., Ahmad H., Rehman K., Keen M., Naveed F., Jawad H., Ikram J. (2025). Hematological malignancies in the United States: What do mortality trends reveal?. J. Clin. Oncol..

[3] Sun K., Wu H., Zhu Q., Gu K., Wei H., Wang S., Li L., Wu C., Chen R., Pang Y. (2025). Global landscape and trends in lifetime risks of haematologic malignancies in 185 countries: Population-based estimates from GLOBOCAN 2022. EClinicalMedicine.

[4] Kumar S.K., Rajkumar V., Kyle R.A., van Duin M., Sonneveld P., Mateos M.-V., Gay F., Anderson K.C. (2017). Multiple myeloma. Nat. Rev. Dis. Primers.

[5] de Pellegars-Malhortie A., Picque Lasorsa L., Mazard T., Granier F., Prévostel C. (2024). Why Is Wnt/β-Catenin Not Yet Targeted in Routine Cancer Care?. Pharmaceuticals.

[6] Frenquelli M., Tonon G. (2020). WNT Signaling in Hematological Malignancies. Front. Oncol..

[7] Clevers H. (2006). Wnt/beta-catenin signaling in development and disease. Cell.

[8] Fergany A., Hassanein K.M., Hameed M.R.A., Kamel A.M., Zahran A.M. (2025). CD34+ CD38-stem cells and CD34+ CD38+ progenitor cells as markers of chemotherapy response in acute myeloid leukemia patients. Immunopathol. Persa.

[9] Yuan Y., Guo M., Gu C., Yang Y. (2021). The role of Wnt/β-catenin signaling pathway in the pathogenesis and treatment of multiple myeloma (review). Am. J. Transl. Res..

[10] Prossomariti A., Piazzi G., Alquati C., Ricciardiello L. (2020). Are Wnt/β-Catenin and PI3K/AKT/mTORC1 Distinct Pathways in Colorectal Cancer?. Cell. Mol. Gastroenterol. Hepatol..

[11] Wu X., Que H., Li Q., Wei X. (2025). Wnt/β-catenin mediated signaling pathways in cancer: Recent advances, and applications in cancer therapy. Mol. Cancer.

[12] Tian H., Wang P., Shao H. (2025). Natural Bioactive Compounds Targeting the Wnt/β-Catenin Pathway for the Treatment of Hepatocellular Carcinoma. J. Hepatocell. Carcinoma.

[13] Xue C., Chu Q., Shi Q., Zeng Y., Lu J., Li L. (2025). Wnt signaling pathways in biology and disease: Mechanisms and therapeutic advances. Signal Transduct. Target. Ther..

[14] van Amerongen R., Nusse R. (2009). Towards an integrated view of Wnt signaling in development. Development.

[15] Zhan T., Rindtorff N., Boutros M. (2017). Wnt signaling in cancer. Oncogene.

[16] Waheed H.J., Abbas W.A.K. (2025). Assessment of serum CLEC4M and glutathione levels in newly diagnosed cervical cancer patients in Iraq: A case-control study. Immunopathol. Persa.

[17] Kahn M. (2014). Can we safely target the WNT pathway?. Nat. Rev. Drug Discov..

[18] Nejad H.H., Hossein F.N., Kamalvand P., Hosseini S.A., Farrokhpour M. (2025). The role of immunogenomics in advancing precision immunotherapy for non-small cell lung cancer. Immunopathol. Persa.

[19] Gajos-Michniewicz A., Czyz M. (2020). WNT Signaling in Melanoma. Int. J. Mol. Sci..

[20] He S., Tang S. (2020). WNT/β-catenin signaling in the development of liver cancers. Biomed. Pharmacother..

[21] Zerehpoosh F.B., Farahmandfar M., Sharifi G., Rezaei O., Gachkar L. (2025). Immunohistochemical evaluation of CD10, BCL6, BCL2, MUM1 and MYC in diffuse large B-cell brain lymphoma; diagnostic and prognostic significance. Immunopathol. Persa.

[22] Niehrs C., Shen J. (2010). Regulation of Lrp6 phosphorylation. Cell. Mol. Life Sci..

[23] Cadigan K.M., Waterman M.L. (2012). TCF/LEFs and Wnt signaling in the nucleus. Cold Spring Harb. Perspect. Biol..

[24] Butler M.T., Wallingford J.B. (2017). Planar cell polarity in development and disease. Nat. Rev. Mol. Cell Biol..

[25] De A. (2011). Wnt/Ca^2+^ signaling pathway: A brief overview. Acta Biochim. Biophys. Sin..

[26] Cruciat C.M., Niehrs C. (2013). Secreted and transmembrane wnt inhibitors and activators. Cold Spring Harb. Perspect. Biol..

[27] Ferreira J.M., Gonçalves C.S., Costa B.M. (2024). Emerging roles and biomarker potential of WNT6 in human cancers. Cell Commun. Signal..

[28] Tufail M., Jiang C.-H., Li N. (2025). Wnt signaling in cancer: From biomarkers to targeted therapies and clinical translation. Mol. Cancer.

[29] Wei M., Zhang C., Tian Y., Du X., Wang Q., Zhao H. (2020). Expression and function of WNT6: From development to disease. Front. Cell Dev. Biol..

[30] Zheng X., Yu H. (2018). Wnt6 contributes tumorigenesis and development of colon cancer via its effects on cell proliferation, apoptosis, cell-cycle and migration. Oncol. Lett..

[31] Tsukiyama T., Koo B.K., Hatakeyama S. (2021). Post-translational Wnt receptor regulation: Is the fog slowly clearing? The molecular mechanism of RNF43/ZNRF3 ubiquitin ligases is not yet fully elucidated and still controversial. Bioessays.

[32] Chartier C., Raval J., Axelrod F., Bond C., Cain J., Dee-Hoskins C., Ma S., Fischer M.M., Shah J., Wei J. (2016). Therapeutic targeting of tumor-derived R-spondin attenuates β-catenin signaling and tumorigenesis in multiple cancer types. Cancer Res..

[33] Carpenter K.A., Thurlow K.E., Craig S.E.L., Grainger S. (2023). Wnt regulation of hematopoietic stem cell development and disease. Curr. Top. Dev. Biol..

[34] Brown G. (2022). Oncogenes and the Origins of Leukemias. Int. J. Mol. Sci..

[35] Wagstaff M., Coke B., Hodgkiss G.R., Morgan R.G. (2022). Targeting β-catenin in acute myeloid leukaemia: Past, present, and future perspectives. Biosci. Rep..

[36] Evangelisti C., Chiarini F., Cappellini A., Paganelli F., Fini M., Santi S., Martelli A.M., Neri L.M., Evangelisti C. (2020). Targeting Wnt/β-catenin and PI3K/Akt/mTOR pathways in T-cell acute lymphoblastic leukemia. J. Cell. Physiol..

[37] Chiarini F., Paganelli F., Martelli A.M., Evangelisti C. (2020). The Role Played by Wnt/β-Catenin Signaling Pathway in Acute Lymphoblastic Leukemia. Int. J. Mol. Sci..

[38] Tickenbrock L., Hehn S., Sargin B., Choudhary C., Bäumer N., Buerger H., Schulte B., Müller O., Berdel W.E., Müller-Tidow C. (2008). Activation of Wnt signalling in acute myeloid leukemia by induction of Frizzled-4. Int. J. Oncol..

[39] Simon M., Grandage V.L., Linch D.C., Khwaja A. (2005). Constitutive activation of the Wnt/beta-catenin signalling pathway in acute myeloid leukaemia. Oncogene.

[40] Feder K., Edmaier-Schröger K., Rawat V.P.S., Kirsten N., Metzeler K., Kraus J.M., Döhner K., Döhner H., Kestler H.A., Feuring-Buske M. (2020). Differences in expression and function of LEF1 isoforms in normal versus leukemic hematopoiesis. Leukemia.

[41] Hallek M. (2025). Chronic Lymphocytic Leukemia: 2025 Update on the Epidemiology, Pathogenesis, Diagnosis, and Therapy. Am. J. Hematol..

[42] Lu D., Liu J.X., Endo T., Zhou H., Yao S., Willert K., Schmidt-Wolf I.G., Kipps T.J., Carson D.A. (2009). Ethacrynic acid exhibits selective toxicity to chronic lymphocytic leukemia cells by inhibition of the Wnt/beta-catenin pathway. PLoS ONE.

[43] Caliskan C., Yuce Z., Ogun Sercan H. (2023). Dvl proteins regulate SMAD1, AHR, mTOR, BRD7 protein expression while differentially regulating canonical and non-canonical Wnt signaling pathways in CML cell lines. Gene.

[44] Sun C., Luan S., Zhang G., Wang N., Shao H., Luan C. (2017). CEBPA-mediated upregulation of the lncRNA PLIN2 promotes the development of chronic myelogenous leukemia via the GSK3 and Wnt/β-catenin signaling pathways. Am. J. Cancer Res..

[45] Pan Y., Chen K., Liang L., Li M., Pan Y. (2024). The Diseases of Hematopoietic and Lymphoid Systems. Textbook of Pathologic Anatomy: For Medical Students.

[46] Hu Y., Zhao Y., Shi C., Ren P., Wei B., Guo Y., Ma J. (2019). A circular RNA from APC inhibits the proliferation of diffuse large B-cell lymphoma by inactivating Wnt/β-catenin signaling via interacting with TET1 and miR-888. Aging.

[47] Wang H., Liu Z., Zhang G. (2020). FBN1 promotes DLBCL cell migration by activating the Wnt/β-catenin signaling pathway and regulating TIMP1. Am. J. Transl. Res..

[48] Zhou H., Tang H., Li N., Chen H., Chen X., Gu L., Zhang L., Tian G., Tao D. (2020). MicroRNA-361-3p Inhibit the Progression of Lymphoma by the Wnt/β-Catenin Signaling Pathway. Cancer Manag. Res..

[49] Belachew A.A., Wu X., Callender R., Waller R., Orlowski R.Z., Vachon C.M., Camp N.J., Ziv E., Hildebrandt M.A.T. (2021). Genetic determinants of multiple myeloma risk within the Wnt/beta-catenin signaling pathway. Cancer Epidemiol..

[50] Rizzieri D.A., Cooley S., Odenike O., Moonan L., Chow K.H., Jackson K., Wang X., Brail L., Borthakur G. (2016). An open-label phase 2 study of glycogen synthase kinase-3 inhibitor LY2090314 in patients with acute leukemia. Leuk. Lymphoma.

[51] Lee J.H., Faderl S., Pagel J.M., Jung C.W., Yoon S.S., Pardanani A.D., Becker P.S., Lee H., Choi J., Lee K. (2020). Phase 1 study of CWP232291 in patients with relapsed or refractory acute myeloid leukemia and myelodysplastic syndrome. Blood Adv..

[52] You H., Wang D., Wei L., Chen J., Li H., Liu Y. (2022). Deferoxamine Inhibits Acute Lymphoblastic Leukemia Progression through Repression of ROS/HIF-1α, Wnt/β-Catenin, and p38MAPK/ERK Pathways. J. Oncol..

[53] Lu D., Choi M.Y., Yu J., Castro J.E., Kipps T.J., Carson D.A. (2011). Salinomycin inhibits Wnt signaling and selectively induces apoptosis in chronic lymphocytic leukemia cells. Proc. Natl. Acad. Sci. USA.

[54] Nadeem B.B., Bibi A., Khan M., Sajjad G.R., Adnan F., Ahmad Z., Khan D. (2024). Effects of imidazole derivatives on cellular proliferation and apoptosis in myeloid leukemia. BMC Cancer.

[55] Lv Z., Wu X., Lu P., Xu X., Wang J., Zhang C., Liu W., Gao Y., Lu C., Zhang Y. (2024). POLE2 knockdown suppresses lymphoma progression via downregulating Wnt/β-catenin signaling pathway. Mol. Cell. Biochem..

[56] Xu J., Lu H., Shi Y., Lei Y., Li X., Cheng W. (2025). Dujieqing decoction suppresses multiple myeloma growth by inhibiting the Wnt/β-catenin pathway. J. Tradit. Chin. Med..

[57] Cromm P.M., Samarasinghe K.T., Hines J., Crews C.M. (2018). Addressing kinase-independent functions of Fak via PROTAC-mediated degradation. J. Am. Chem. Soc..

[58] Trapani J., Caroland K.P., Ahmed Y., Robbins D.J., Weiss V.L., Lee E. (2026). Targeting β-catenin: PROTACs and precision degraders for Wnt-driven cancers. Front. Oncol..

[59] Hadate Y., Hattori Y., Toda Y., Hosogi S., Okada S., Hayashi Y., Ashihara E. (2024). Compound #41 Targets Acute Myelogenous Leukemia by Inhibiting the Wnt/β-catenin Signaling Pathway. Anticancer. Res..

[60] Gao Q., Hou L., Wang H., Xun L. (2022). DACT3 has a tumor-inhibiting role in acute myeloid leukemia via the suppression of Wnt/β-catenin signaling by DVL2. J. Biochem. Mol. Toxicol..

[61] Shahid A.M., Um I.H., Elshani M., Zhang Y., Harrison D.J. (2022). NUC-7738 regulates β-catenin signalling resulting in reduced proliferation and self-renewal of AML cells. PLoS ONE.

[62] Mao P., Huang C., Li Y., Zhao Y., Zhou S., Zhao Z., Mu Y., Wang L., Li F., Zhao A.Z. (2023). Pharmacological targeting of type phosphodiesterase 4 inhibits the development of acute myeloid leukemia by impairing mitochondrial function through the Wnt/β-catenin pathway. Biomed. Pharmacother..

[63] Lei Y., Li X., Zhu L. (2024). Matrine regulates miR-495-3p/miR-543/PDK1 axis to repress the progression of acute myeloid leukemia via the Wnt/β-catenin pathway. Chem. Biol. Drug Des..

[64] Al-Hamaly M.A., Cox A.H., Haney M.G., Zhang W., Arvin E.C., Sampathi S., Wimsett M., Liu C., Blackburn J.S. (2024). Zebrafish drug screening identifies Erlotinib as an inhibitor of Wnt/β-catenin signaling and self-renewal in T-cell acute lymphoblastic leukemia. Biomed. Pharmacother..

[65] Zhao L., Lv C., Sun L., Li Q., Wang Y., Wu M., Wang Y., Guo Z., Bian S., Kong D. (2021). Histone deacetylase inhibitor chidamide regulates the Wnt/β-catenin pathway by MYCN/DKK3 in B-ALL. Investig. New Drugs.

[66] Li W., Zhao Y., Qiu L., Ma J. (2019). Effect of Quercetin on Wnt/β-Catenin Signal Pathway of K562 and K562R Cells. Zhongguo Shi Yan Xue Ye Xue Za Zhi.

[67] Hong L., Yilin W., Caili W., Xiaomin W., Min M., Yan L. (2025). The research on the mechanism of microRNA-34a influencing the progression of chronic lymphocytic leukemia by regulating the Wnt pathway. Tianjin Med. J..

[68] Gandhirajan R.K., Staib P.A., Minke K., Gehrke I., Plickert G., Schlösser A., Schmitt E.K., Hallek M., Kreuzer K.A. (2010). Small molecule inhibitors of Wnt/beta-catenin/lef-1 signaling induces apoptosis in chronic lymphocytic leukemia cells in vitro and in vivo. Neoplasia.

[69] Li L., Zeng P., Yu L., Yang J., Man J., Zhou L., Zhao L. (2023). Salinomycin sodium exerts anti diffuse large B-cell lymphoma activity through inhibition of LRP6-mediated Wnt/β-catenin and mTORC1 signaling. Leuk. Lymphoma.

[70] Zhang Y., Wang C.P., Ding X.X., Wang N., Ma F., Jiang J.H., Wang Q.D., Chang J.B. (2014). FNC, a novel nucleoside analogue, blocks invasion of aggressive non-Hodgkin lymphoma cell lines via inhibition of the Wnt/β-catenin signaling pathway. Asian Pac. J. Cancer Prev..

[71] Yao H., Ashihara E., Strovel J.W., Nakagawa Y., Kuroda J., Nagao R., Tanaka R., Yokota A., Takeuchi M., Hayashi Y. (2011). AV-65, a novel Wnt/β-catenin signal inhibitor, successfully suppresses progression of multiple myeloma in a mouse model. Blood Cancer J..

[72] Zeng D., Liu M., Pan J. (2017). Blocking EZH2 methylation transferase activity by GSK126 decreases stem cell-like myeloma cells. Oncotarget.

[73] Wang H., Gong Y., Liang L., Xiao L., Yi H., Ye M., Roy M., Xia J., Zhou W., Yang C. (2020). Lycorine targets multiple myeloma stem cell-like cells by inhibition of Wnt/β-catenin pathway. Br. J. Haematol..

[74] Wei Z., Wang W., Li Q., Du L., He X. (2021). The microRNA miR-19a-3p suppresses cell growth, migration, and invasion in multiple myeloma via the Wnt/β-catenin pathway. Transl. Cancer Res..

[75] Wang H., Xiao X., Li Z., Luo S., Hu L., Yi H., Xiang R., Zhu Y., Wang Y., Zhu L. (2022). Polyphyllin VII, a novel moesin inhibitor, suppresses cell growth and overcomes bortezomib resistance in multiple myeloma. Cancer Lett..

[76] Choi P.J., O Y., Her J.H., Yun E., Song G.Y., Oh S. (2017). Anti-proliferative activity of CGK012 against multiple myeloma cells via Wnt/β-catenin signaling attenuation. Leuk. Res..

[77] Liu X., Peng J., Zhou Y., Xie B., Wang J. (2019). Silencing RRM2 inhibits multiple myeloma by targeting the Wnt/β-catenin signaling pathway. Mol. Med. Rep..

[78] Son Y., Quan K.T., Shin S., Park S., Na M., Oh S. (2022). Lucidin 3-methyl ether from Rubia philippinensis suppresses the proliferation of multiple myeloma cells through the promotion of β-catenin degradation. Phytomedicine.

[79] Savvidou I., Khong T., Cuddihy A., McLean C., Horrigan S., Spencer A. (2017). β-Catenin Inhibitor BC2059 Is Efficacious as Monotherapy or in Combination with Proteasome Inhibitor Bortezomib in Multiple Myeloma. Mol. Cancer Ther..

[80] Kim S.J., Kim S., Choi Y.J., Kim U.J., Kang K.W. (2022). CKD-581 Downregulates Wnt/β-Catenin Pathway by DACT3 Induction in Hematologic Malignancy. Biomol. Ther..

[81] Pan M., Jiao C., Sun M., Jin D., Wang Y., Wu H., Zhang Y., Chen E., Su B., Zhou J. (2025). Venetoclax synergizes with Wnt/β-catenin inhibitor C-82 in acute myeloid leukemia by increasing the degradation of Mcl-1 protein. Cancer Cell Int..

[82] Zhao L., Sun L., Kong D., Cao R., Guo Z., Guo D., Li Q., Hao J., Li Y., Emails L. (2025). Chidamide and venetoclax synergistically regulate the Wnt/β-catenin pathway by MYCN/DKK3 in B-ALL. Ann. Hematol..

[83] Ding L., Chen Q., Chen K., Jiang Y., Li G., Chen Q., Bai D., Gao D., Deng M., Zhang H. (2021). Simvastatin potentiates the cell-killing activity of imatinib in imatinib-resistant chronic myeloid leukemia cells mainly through PI3K/AKT pathway attenuation and Myc downregulation. Eur. J. Pharmacol..

[84] Han Y., Li C., Liu S., Gao J., He Y., Xiao H., Chen Q., Zheng Y., Chen H., Zhu X. (2024). Combined targeting of Hedgehog/GLI1 and Wnt/β-catenin pathways in mantle cell lymphoma. Hematol. Oncol..

[85] Higuchi Y., Teo J.-L., Yi D., Kahn M. (2025). Safely Targeting Cancer, the Wound That Never Heals, Utilizing CBP/Beta-Catenin Antagonists. Cancers.

[86] Che M., Kweon S.-M., Teo J.-L., Yuan Y.-C., Melstrom L.G., Waldron R.T., Lugea A., Urrutia R.A., Pandol S.J., Lai K.K. (2020). Targeting the CBP/β-catenin interaction to suppress activation of cancer-promoting pancreatic stellate cells. Cancers.

[87] Marr A.R., Halpin M., Corbin D.L., Asemelash Y., Sher S., Gordon B.K., Whipp E.C., Mitchell S., Harrington B.K., Orwick S. (2024). The multi-CDK inhibitor dinaciclib reverses bromo- and extra-terminal domain (BET) inhibitor resistance in acute myeloid leukemia via inhibition of Wnt/β-catenin signaling. Exp. Hematol. Oncol..

[88] Wakabayashi R., Hattori Y., Hosogi S., Toda Y., Takata K., Ashihara E. (2021). A novel dipeptide type inhibitor of the Wnt/β-catenin pathway suppresses proliferation of acute myelogenous leukemia cells. Biochem. Biophys. Res. Commun..

[89] Jiang X., Mak P.Y., Mu H., Tao W., Mak D.H., Kornblau S., Zhang Q., Ruvolo P., Burks J.K., Zhang W. (2018). Disruption of Wnt/β-Catenin Exerts Antileukemia Activity and Synergizes with FLT3 Inhibition in FLT3-Mutant Acute Myeloid Leukemia. Clin. Cancer Res..

[90] Xi H.M., Lu H., Weng X.Q., Sheng Y., Wu J., Li L., Cai X. (2023). Combined Application of Salinomycin and ATRA Induces Apoptosis and Differentiation of Acute Myeloid Leukemia Cells by Inhibiting WNT/β-Catenin Pathway. Anticancer. Agents Med. Chem..

[91] Fiskus W., Sharma S., Saha S., Shah B., Devaraj S.G., Sun B., Horrigan S., Leveque C., Zu Y., Iyer S. (2015). Pre-clinical efficacy of combined therapy with novel β-catenin antagonist BC2059 and histone deacetylase inhibitor against AML cells. Leukemia.

[92] Takam Kamga P., Dal Collo G., Cassaro A., Bazzoni R., Delfino P., Adamo A., Bonato A., Carbone C., Tanasi I., Bonifacio M. (2020). Small Molecule Inhibitors of Microenvironmental Wnt/β-Catenin Signaling Enhance the Chemosensitivity of Acute Myeloid Leukemia. Cancers.

[93] Fu J., Si L., Zhuang Y., Zhang A., Sun N., Li D., Hao B., Ju X. (2019). Wnt/β-catenin inhibition reverses multidrug resistance in pediatric acute lymphoblastic leukemia. Oncol. Rep..

[94] Kumrić M., Tičinović Kurir T., Borovac J.A., Božić J. (2020). The Role of Natural Killer (NK) Cells in Acute Coronary Syndrome: A Comprehensive Review. Biomolecules.

[95] Zhang H., Wang Y., Yang H., Huang Z., Wang X., Feng W. (2021). TCF7 knockdown inhibits the imatinib resistance of chronic myeloid leukemia K562/G01 cells by neutralizing the Wnt/β-catenin/TCF7/ABC transporter signaling axis. Oncol. Rep..

[96] Zhou H., Mak P.Y., Mu H., Mak D.H., Zeng Z., Cortes J., Liu Q., Andreeff M., Carter B.Z. (2017). Combined inhibition of β-catenin and Bcr-Abl synergistically targets tyrosine kinase inhibitor-resistant blast crisis chronic myeloid leukemia blasts and progenitors in vitro and in vivo. Leukemia.

[97] Bai M., Huang Y., Suo X., Wang L., Han W., Zhang W. (2024). BET bromodomain inhibitors PFI-1 and CPI-203 suppress the development of follicular lymphoma via regulating Wnt/β-catenin signaling. Heliyon.

[98] Wu C. (2022). β-catenin inhibitors ICG-001 and pyrvinium sensitize bortezomib-resistant multiple myeloma cells to bortezomib. Oncol. Lett..

[99] Jin Y., Xu L., Wu X., Feng J., Shu M., Gu H., Gao G., Zhang J., Dong B., Chen X. (2019). Synergistic Efficacy of the Demethylation Agent Decitabine in Combination With the Protease Inhibitor Bortezomib for Treating Multiple Myeloma Through the Wnt/β-Catenin Pathway. Oncol. Res..

[100] Cui C., Zhou X., Zhang W., Qu Y., Ke X. (2018). Is β-Catenin a Druggable Target for Cancer Therapy?. Trends Biochem. Sci..

[101] Baron R., Rawadi G. (2007). Targeting the Wnt/beta-catenin pathway to regulate bone formation in the adult skeleton. Endocrinology.

[102] Feng L., Tian R., Mu X., Chen C., Zhang Y., Cui J., Song Y., Liu Y., Zhang M., Shi L. (2022). Identification of Genes Linking Natural Killer Cells to Apoptosis in Acute Myocardial Infarction and Ischemic Stroke. Front. Immunol..

[103] Choi B.Y. (2020). Targeting Wnt/β-Catenin Pathway for Developing Therapies for Hair Loss. Int. J. Mol. Sci..

[104] Russell J.O., Monga S.P. (2018). Wnt/β-Catenin Signaling in Liver Development, Homeostasis, and Pathobiology. Annu. Rev. Pathol..

[105] Singh G., Hossain M.M., Bhat A.Q., Ayaz M.O., Bano N., Eachkoti R., Dar M.J. (2021). Identification of a cross-talk between EGFR and Wnt/beta-catenin signaling pathways in HepG2 liver cancer cells. Cell. Signal..

[106] Lin Y., Higashisaka K., Shintani T., Maki A., Hanamuro S., Haga Y., Maeda S., Tsujino H., Nagano K., Fujio Y. (2020). Progesterone receptor membrane component 1 leads to erlotinib resistance, initiating crosstalk of Wnt/β-catenin and NF-κB pathways, in lung adenocarcinoma cells. Sci. Rep..

[107] Daisy Precilla S., Biswas I., Kuduvalli S.S., Anitha T.S. (2022). Crosstalk between PI3K/AKT/mTOR and WNT/β-Catenin signaling in GBM—Could combination therapy checkmate the collusion?. Cell. Signal..

[108] Liu C., Shen A., Song J., Cheng L., Zhang M., Wang Y., Liu X. (2024). LncRNA-CCAT5-mediated crosstalk between Wnt/β-Catenin and STAT3 signaling suggests novel therapeutic approaches for metastatic gastric cancer with high Wnt activity. Cancer Commun..

[109] Yu F., Yu C., Li F., Zuo Y., Wang Y., Yao L., Wu C., Wang C., Ye L. (2021). Wnt/β-catenin signaling in cancers and targeted therapies. Signal Transduct. Target. Ther..

[110] Zhong Z., Virshup D.M. (2020). Wnt signaling and drug resistance in cancer. Mol. Pharmacol..

[111] Song P., Gao Z., Bao Y., Chen L., Huang Y., Liu Y., Dong Q., Wei X. (2024). Wnt/β-catenin signaling pathway in carcinogenesis and cancer therapy. J. Hematol. Oncol..

[112] Li C., Liang Y., Cao J., Zhang N., Wei X., Tu M., Xu F., Xu Y. (2019). The Delivery of a Wnt Pathway Inhibitor Toward CSCs Requires Stable Liposome Encapsulation and Delayed Drug Release in Tumor Tissues. Mol. Ther..

[113] Arend R., Dholakia J., Castro C., Matulonis U., Hamilton E., Jackson C.G., LyBarger K., Goodman H.M., Duska L.R., Mahdi H. (2023). DKK1 is a predictive biomarker for response to DKN-01: Results of a phase 2 basket study in women with recurrent endometrial carcinoma. Gynecol. Oncol..

[114] Phillips C., Bhamra I., Eagle C., Flanagan E., Armer R., Jones C.D., Bingham M., Calcraft P., Edmenson Cook A., Thompson B. (2022). The Wnt Pathway Inhibitor RXC004 Blocks Tumor Growth and Reverses Immune Evasion in Wnt Ligand-dependent Cancer Models. Cancer Res. Commun..

[115] Delkash P., Parsa A., Abbasi Z., Kavand S., Homaee S., Hadizade F., Saberian F., Dehghani-ghorbi M. (2026). Assessment of L-carnitine as a therapeutic agent for cancer-related fatigue in patients receiving platinum-based chemotherapy regimens; a parallel-group, randomized, double-blind clinical trial study. J. Prev. Epidemiol..

